# Intensive physical activity following total hip arthroplasty increased the revision risk after 15 years: a cohort study of 973 patients from the Geneva Arthroplasty Register

**DOI:** 10.2340/17453674.2024.41192

**Published:** 2024-08-15

**Authors:** Elena ZABALLA, Stefania D’ANGELO, Christophe BAREA, Georgia NTANI, Didier HANNOUCHE, Cyrus COOPER, Anne LÜBBEKE, Karen WALKER-BONE

**Affiliations:** 1 MRC Lifecourse Epidemiology Centre, University of Southampton, Southampton, UK; 2 MRC Versus Arthritis Centre for Musculoskeletal Health and Work, University of Southampton, Southampton, UK; 3 Division of Orthopaedic Surgery and Traumatology, Geneva University Hospitals and University of Geneva, Geneva, Switzerland; 4 Nuffield Department of Orthopaedics, Rheumatology and Musculoskeletal Sciences, University of Oxford, Oxford, UK; 5 Monash Centre for Occupational and Environmental Health, Monash University, Melbourne, Australia

## Abstract

**Background and purpose:**

Younger recipients of total hip arthroplasty (THA) highly prioritize returning to preoperative levels of physical activity (PA). Surgeons have tended to give cautious advice concerning high-impact sports participation, but there have been few long-term studies. The purpose of our study was to investigate the risk of revision arthroplasty in relation to postoperative PA levels.

**Methods:**

Patients registered in the Geneva Arthroplasty Register (GAR) who had elective THA when they were aged < 65 years were studied. Postoperative PA was collected prospectively 5-yearly using the UCLA activity scale. Cox proportional hazards models were used to estimate associations between PA and risk of revision THA.

**Results:**

Amongst 1,370 eligible subjects, median age at THA 58 years (interquartile range 51–61), UCLA scores were available for 973 (71%). During follow-up over 15 years, there were 79 revisions, giving a cumulative risk of 7.4% (95% confidence interval [CI] 5.8–9.4). After adjusting for covariates, we found an increased risk of revision for each unit increase in postoperative PA (HR 1.2, CI 1.1–1.4), and among people performing the most intensive PA (HR 2.7, CI 1.3–5.6) compared with those who were inactive.

**Conclusion:**

The overall risk of revision was small but intensive and moderate PA may be associated with an increased risk of revision.

Given its proven effectiveness at reducing pain and restoring function, total hip arthroplasty (THA) is increasingly offered to younger patients [1]. Younger patients have increasing expectations after THA, including resuming preoperative levels of physical activity (PA). Expectations appear highest amongst those who are most active preoperatively [2] and 80% of recipients return to similar [3] or the same presymptomatic level of sports [4], and 70% return to the same job after arthroplasty.

Orthopedic surgeons may have remained cautious when advising patients on participation in postoperative activities particularly high-impact sports, although their recommendations have become less restrictive over the years [5], perhaps as a result of patients’ experiences and/or decreasing failure rates over time [6]. We aimed to compare the association between postoperative exposure to PA and revision for any cause except infection in elective THA recipients aged < 65 years at time of surgery using registry data over 15 years of follow-up.

## Methods

### Setting

All patients who have undergone primary THA or a hip revision at the Geneva University Hospitals since 1996 have been registered in the Geneva Hip Arthroplasty Registry (GAR). The Registry collected the following information at the time of THA: sex, age, body mass index (BMI), smoking status, American Society of Anesthesiologists (ASA) score, and Charnley score. Interoperatively, date and main indication for THA (primary or secondary osteoarthritis [OA]), type of fixation (cemented, uncemented, hybrid), and size of the femoral head were recorded (22, 28, 32, 36, > 36 mm) as well as type of bearing surfaces (classified as metal-on-metal (MoM) and non-MoM implants (ceramic–highly crosslinked polyethylene, ceramic–polyethylene, metal–polyethylene, and ceramic–ceramic).

Patients were eligible for inclusion in this analysis if: (i) they had undergone elective and unilateral primary THA when they were between 18 and 64 years old, and (ii) their surgery was performed anytime between inception of GAR and December 2012. A prior decision was taken to exclude patients (i) who had undergone hip resurfacing, (ii) who received an emergency THA (i.e., a non-elective procedure), and (iii) who had an implanted femoral head > 36 mm, due to the high revision rate associated with these risk factors. Additionally, revisions carried out within 6 months of the index surgery (more likely to be indicated by either intraoperative factors or early infections) were also excluded. Given the length of postoperative follow-up, some participants had 2 hips replaced, in which case the first THA recorded in GAR was taken as the index case.

Since April 2006, postoperative PA levels have been collected 5-yearly using the University of California, Los Angeles [7] (UCLA) activity scale [8]. Patients are asked to choose the level of activity that best represents their current PA on a scale from 0 (wholly inactive, dependent on others, cannot leave residence) to 10 (very active, regularly participate in impact sports). Due to differences in the length of follow-up between subjects, and because some patients had UCLA scale available at multiple follow-ups, we calculated a mean UCLA score for each person. Given a lack of consensus as to how to classify PA as, e.g., high, moderate, or low, the following pragmatic categories were used: low activity group (UCLA 1–4), moderate activity group (UCLA 5–7) and high activity group (UCLA 8–10).

### Outcome

The outcome was revision of the primary THA for any reason other than infection. For the latter group, the time patients contributed was also censored at the date of revision. Both prosthesis removal and replacement, and removal and replacement of any of the components of the prosthesis such as cups or stems, were considered a revision. In July 2022, we extracted data from the registry, and we were able to confirm the status of each participant.

### Statistics

First, the baseline characteristics of THA recipients with and without UCLA scale were compared, using counts and percentages for categorical variables and medians and interquartile ranges (IQR) for continuous non-normally distributed variables. Then, for participants with UCLA scale, baseline characteristics were compared across levels of UCLA (low/moderate/high). Standardized differences between means or prevalences across all pairwise combinations of UCLA categories were also reported [9].

Time at risk of revision (in years) was computed as the time between THA surgery and the earliest of: death, loss to follow-up, revision surgery (if the participant experienced the event of interest), or end of follow-up (July 1, 2022) if no event was experienced. Prevalence rates and mean time contributed per person were then calculated for PA as both a continuous and categorical variable.

To illustrate THA failures over time, we calculated cumulative risks of revision and their 95% confidence intervals (CI) at 10 and 15 years postoperatively, and we constructed Kaplan–Meier failure plots with 95% CI and smooth hazard curves by categories of postoperative PA (low, moderate, and high), and also by bearing surface of the implant.

To assess the association between the risk of THA revision and PA post arthroplasty, Cox proportional hazard regression models were fitted with 95% confidence intervals (CI) before and after adjusting for sex, age at the time of primary operation, and type of bearing surface. We tested the assumption of the proportional-hazards model and whether there was an interaction effect between PA and type of bearing surface, given the higher rate of failure in MoM implants [10]. All analyses were performed using Stata version 17.0 (StataCorp LLC, College Station, TX, USA).

### Ethics, registration, data sharing, funding, use of AI, and disclosures

Ethics approval to perform this study was obtained from the Commission cantonale d’éthique de la recherche (CCER), number PB_2017-00164 (05-2017).

Data are available on reasonable request. Researchers requesting access to Geneva Arthroplasty Registry data may contact AL (anne.lubbekewolff@hcuge.ch).

The Medical Research Council Versus Arthritis (formerly Arthritis Research UK) Centre for Musculoskeletal Health and Work supported this work (award number 22090). The funder had no role in the study design, data collection, analysis of data, writing the manuscript, or in the decision to submit the manuscript for publication.

The authors declare that no artificial intelligence tools or technologies were used in the study design, data collection, analysis of data, or writing up of this manuscript.

CC has received lecture fees and honoraria from Amgen, Danone, Eli Lilly, GSK, Kyowa Kirin, Medtronic, Merck, Nestlé, Novartis, Pfizer, Roche, Servier, Shire, Takeda, and UCB outside of the submitted work. AL is a member of the steering committee of the International Society of Arthroplasty Registries, and an editorial board member of EFFORT Open Reviews.

The authors declare no conflict of interest in relation to the content of this manuscript. Complete disclosure of interest forms according to ICMJE are available on the article page, doi: 10.2340/17453674.2024.41192

## Results

**Figure 1:**
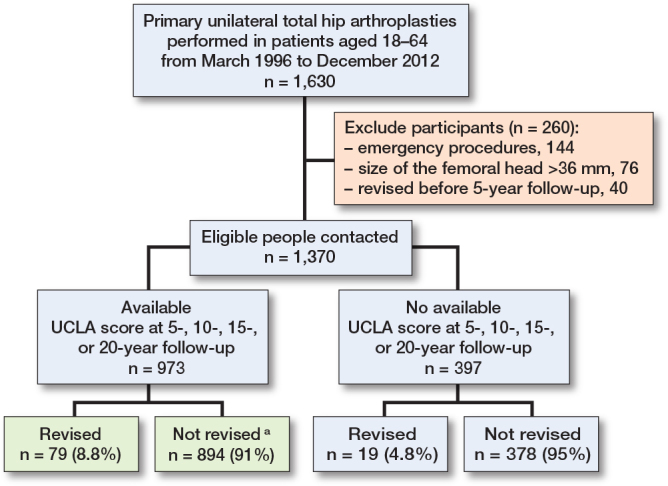
Flowchart describing participants from the Geneva Hip Arthroplasty Registry eligible for this study. **^a^** In the not revised group 7of 894 underwent THA revision for infection or unknown reasons.

1,370 THA recipients fulfilled the inclusion criteria (Figure 1), of whom 973 (71%) had completed the UCLA scale at either 5-, 10-, 15-, and/or 20-year follow-up depending on when the primary operation was performed, and whether they had attended follow-up visits. This meant that patients who underwent revision within 5 years after operation were excluded from the analysis because they did not complete the first UCLA score assessment. 89 surgeons had operated on the participants: 50% of the interventions were done by an experienced THA surgeon, and 50% by surgeons in training under the supervision of an experienced THA surgeon. In total, 79% of these THAs were performed via the lateral approach (Hardinge), 15% via an anterior approach (Hueter), 5% via a posterior approach, and 1% using a trans-trochanteric approach. The vast majority of implants were a standard cup and only 12 patients received a dual-mobility cup.

### Baseline characteristics

Baseline data showed that, compared with non-respondents, those with UCLA scores were more likely to be fitter, as indicated by the ASA score (Table 1, see Appendix). For those who reported UCLA scores, participants who were most physically active were younger, more likely to be of normal weight, non-smokers, fitter at as indicated by the ASA score, and more likely to have a MoM implant (Table 2). Primary OA was the main indication for THA across all 3 groups.

**Table 1:** Characteristics of total hip arthroplasty (THA) recipients operated on between March 1996 and December 2012 comparing those who did and did not complete at least 1 postoperative UCLA score. Values are count (%) unless otherwise specified

Factor	All n = 1,370	No UCLA score after THA (non-respondents) n = 397	UCLA score completed after THA (respondents) n = 973	P value
Age at THA,				
median (IQR)	58 (51–61)	57 (50–61)	58 (52–61)	0.06**^b^**
Male sex	753 (55)	244 (61)	509 (52)	< 0.01**^a^**
Body mass index				
< 18.5	26 (2.0)	14 (3.5)	12 (1.2)	0.2**^a^**
18.5–24.9	445 (32)	133 (34)	312 (32)	
25.0–29.9	515 (38)	138 (35)	377 (39)	
≥ 30.0	378 (28)	106 (27)	272 (28)	
Missing	6 (0.4)	6 (1.5)	0 (0)	
Smoking				
No	830 (61)	208 (52)	622 (64)	< 0.01**^a^**
Yes	452 (33)	146 (37)	306 (31)	
Missing	88 (6.4)	43 (10.8)	45 (4.6)	
Indication for THA				
Primary OA	858 (63)	235 (59)	623 (64)	0.09**^a^**
Secondary OA	512 (37)	162 (41)	350 (36)	
Charnley score				
A	539 (39)	156 (39)	383 (39)	0.3**^c^**
B	470 (34)	124 (31)	346 (36)	
C	353 (26)	116 (29)	237 (24)	
Missing	8 (0.6)	1 (0.3)	7 (0.7)	
ASA score				
1	285 (21)	73 (18)	212 (22)	< 0.01**^c^**
2	954 (70)	262 (66)	692 (71)	
3	129 (9.4)	61 (15)	68 (7.0)	
4	2 (0.1)	1 (0.3)	1 (0.1)	
Fixation				
Uncemented	489 (36)	143 (36)	346 (36)	0.5 **^a^**
Cemented	52 (3.8)	19 (4.8)	33 (3.4)	
Hybrid	829 (61)	235 (59)	594 (61)	
Bearing surfacing				
Other	866 (63)	262 (66)	604 (62)	0.2**^a^**
MoM	504 (37)	135 (34)	369 (38)	
Femoral head size (mm)				
22	11 (0.8)	4 (1.0)	7 (0.7)	0.03**^a^**
28	1159 (85)	323 (81)	836 (86)	
32	124 (9.1)	40 (10)	84 (8.6)	
36	76 (5.5)	30 (8.6)	46 (4.7)	

OA = osteoarthritis ; MoM = metal-on-metal.

Selection bias assessed comparing respondents and non-respondents:

a χ^2^ test,

b Wilcoxon rank sum test,

c Spearman test for trend.

**Table 2:** Characteristics of patients receiving a total hip arthroplasty (THA) by level of physical activity. Values are count (%) unless otherwise specified

Factor	Low UCLA 1–4 n = 271	Moderate UCLA 5–7 n = 520	High UCLA 8–10 n = 182	Standardized difference		
				Low vs moderate	Low vs high	Moderate vs high
Age at THA **^a^**	58 (53–61)	59 (52–62)	56 (48–60)	–0.040	0.269	0.305
Male sex	137 (51)	269 (52)	103 (57)	–0.022	–0.121	–0.098
Body mass index						
< 18.5	3 (1.1)	5 (1.0)	4 (2.2)	0.493	0.363	–0.198
18.5–24.9	68 (25)	164 (32)	80 (44)	–0.142	–0.406	–0.260
25.0–29.9	92 (34)	212 (41)	73 (40)	–0.141	–0.127	0.014
≥ 30.0	108 (40)	139 (27)	25 (14)	0.281	0.617	0.328
Smoking						
No	170 (63)	316 (61)	136 (75)	0.039	–0.261	–0.301
Yes	92 (34)	171 (33)	43 (24)	0.023	0.231	0.208
Missing	9 (3.3)	33 (6.4)	3 (1.7)			
Indication for THA						
Primary OA	153 (56)	347 (67)	123 (68)	–0.211	–0.230	–0.019
Secondary OA	118 (44)	173 (33)	59 (32)	0.211	0.230	0.019
Charnley score						
A	80 (30)	217 (42)	86 (47)	–0.257	–0.372	–0.113
B	90 (33)	190 (37)	66 (36)	–0.069	–0.065	0.004
C	100 (37)	109 (21)	28 (15)	0.356	0.504	0.146
Missing	1 (0.4)	4 (0.8)	2 (1.1)			
ASA score						
1	29 (11)	112 (22)	71 (39)	–0.297	–0.693	–0.039
2	207 (76)	378 (73)	107 (59)	0.083	0.380	0.296
3	34 (13)	30 (5.8)	4 (2.2)	–1.080	–0.250	0.790
4	1 (0.4)	–	–			
Fixation						
Uncemented	92 (34)	169 (33)	85 (47)	0.030	–0.261	–0.291
Cemented	11 (4.1)	20 (3.9)	2 (1.1)	0.041	0.728	0.683
Hybrid	168 (62)	331 (64)	95 (52)	–0.035	0.197	0.232
Bearing surfacing						
Other	185 (68)	329 (63)	90 (49)	0.106	0.389	0.281
MoM	86 (32)	191 (37)	92 (51)	–0.106	–0.389	–0.281
Femoral head size (mm)						
22	2 (0.7)	5 (1.0)	–			
28	225 (83)	463 (89)	148 (81)	–0.174	0.044	0.218
32	22 (8.2)	34 (6.5)	28 (15)	0.393	1.787	1.173
36	22 (8.2)	18 (3.5)	6 (3.3)	1.085	1.141	0.042

a median (interquartile range); OA = osteoarthritis ; MoM = metal-on-metal.

Amongst the 973 people with postoperative UCLA scores, the median age at the time of primary surgery was 58 (IQR 52–61) years. In total, 79 revisions (8.8%) were performed (after excluding any performed for infection). The mean time of follow-up to revision surgery or until participants were censored was 15.7 years (standard deviation [SD] 5). The main indications for revision surgery were aseptic loosening (46% low group, 62% moderate group, 65% high group), granuloma (aseptic lymphocyte-dominant vasculitis-associated lesion) (27% low group, 25% moderate group, 17% high group), periprosthetic fracture (9.1% low group, 4.3% moderate group, 8.7% high group), and recurrent dislocation (18% low group, 4.3% moderate group, 8.7% high group).

### Outcome

**Figure 2:**
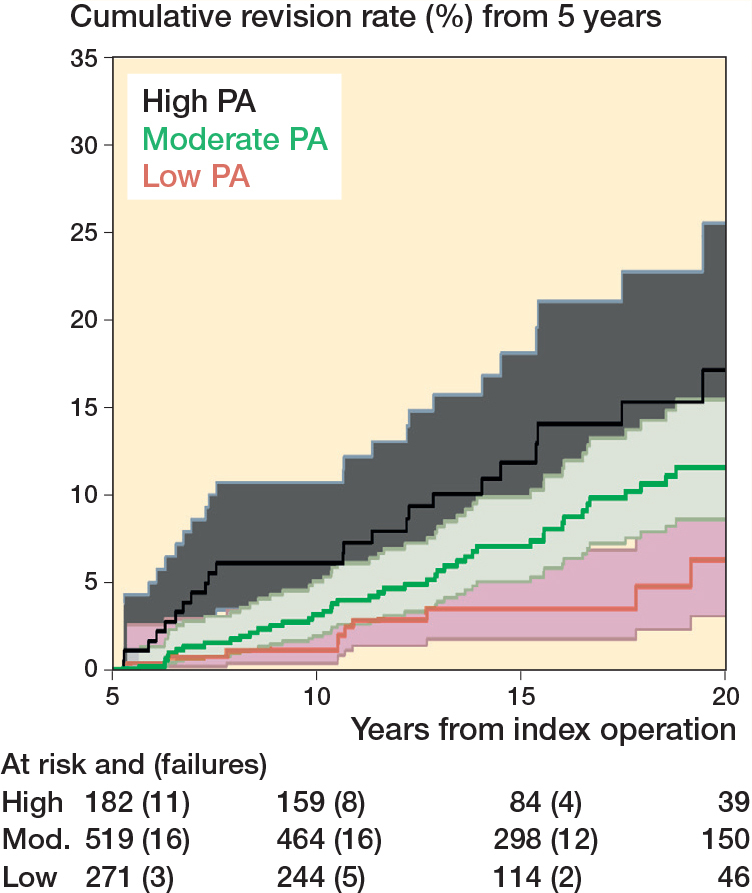
Kaplan–Meier revision events by level of physical activity postoperatively in 973 THA patients. One participant in the Moderate category of physical activity does not feature in the graph as the participant was assessed at 4.9 years from the surgery. 95% CI of moderate and high level of PA overlaps.

**Figure 3:**
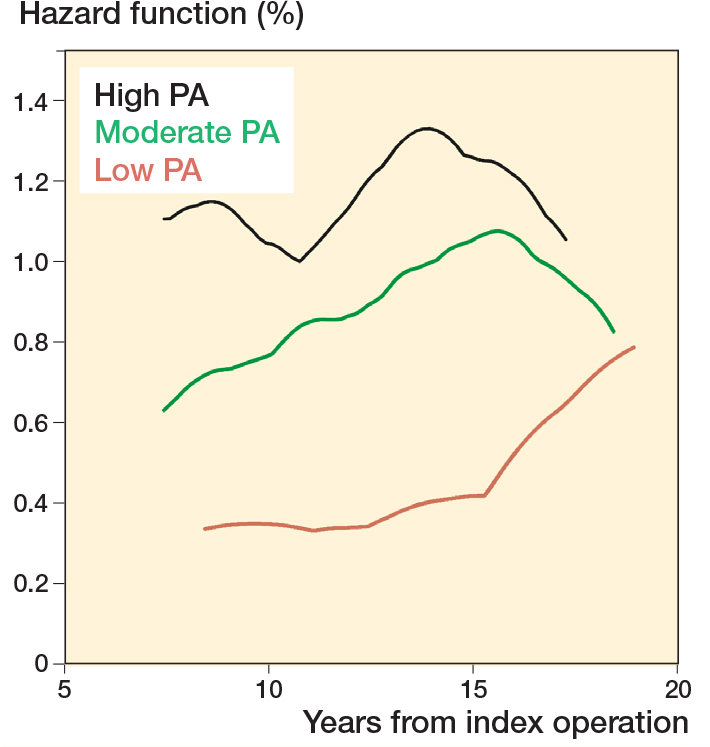
Smoothed hazard estimated by level of physical activity postoperatively in 973 THA patients.

Of the 973 participants, 79 underwent THA revision, 128 died over the course of follow-up, and 52 were lost to follow-up, leaving 707 people at risk of experiencing revision at the end of follow-up (July 2022). Overall, the cumulative risk of revision accounting for censored events was: 3.2% (CI 2.3–4.6) at 10 years and 7.2% (CI 5.6–9.3) at 15 years. By level of PA (low, medium, and high) the risks were: 1.2% (CI 0.4–3.6), 3.2% (CI 2.0–5.2), and 6.3% (CI 3.5–11) at 10 years, and 3.9% (CI 2.0–7.0), 7.2% (CI 5.1–10.0), and 12.2% (CI 7.9–18.6) at 15 years (Table 3). The models for UCLA as a continuous variable showed an increased risk of THA revision for each unit increase in postoperative level of PA (HR 1.2, CI 1.1–1.4). When PA was grouped in 3 categories (low, moderate, and high), there was a 2-fold increased risk of THA revision for those in the highest activity group (HR 2.7, CI 1.3–5.6) and in the moderate activity group (HR 1.9, CI 1.0–3.6) compared with those in the lowest activity group. Higher rates of implant revision were found amongst people who reported more intensive PA post-arthroplasty (Figures 2 and 3).

**Table 3:** Relationship between level of physical activity reported on UCLA score after total hip arthroplasty (THA) and risk of revision in 973 patients who were aged < 65 years at time of index THA

Factor	UCLA activity score continuous	UCLA activity score in 2 categories		UCLA activity score in 3 categories		
		Not high UCLA 1–7	High UCLA 8–10	Low UCLA 1–4	Moderate UCLA 5–7	High UCLA 8–10
Physical activity, mean (SD)						
Not revised	5.8 (1.9)	–	–	–	–	–
Revised	6.6 (1.8)	–	–	–	–	–
Revision, n (%)						
No	–	735 (93)	159 (87)	260 (96)	475 (91)	159 (87)
Yes	–	56 (7)	23 (13)	11 (4.1)	45 (8.7)	23 (13)
Median years to end of study/revision						
Not revised	–	15.8	15.1	13.7	16.8	15.1
Revised	–	11.4	10.6	10.7	11.6	10.6
Models of HR (CI)						
Unadjusted	1.2 (1.1–1.4)	Ref.	1.9 (1.1–3.0)	Ref.	1.9 (1.0–3.6)	3.0 (1.4–6.1)
Adjusted **^a^**	1.2 (1.1–1.4)	Ref.	1.7 (1.0–2.8)	Ref.	1.9 (1.0–3.6)	2.7 (1.3–5.6)

a Adjusted for sex, age at THA, and type of bearing surfacing.

The assumptions of the proportional hazards model were satisfactory, and no interaction was found between type of bearing surface and PA (P = 0.6); nevertheless we reported stratified analysis by type of bearing surfacing (small head MoM and non-MoM) for clinical reasons (Table 4, see Appendix), and plotted Kaplan–Meier failure curves (Supplementary Figure 4, see Appendix). Adjusted models showed that patients with MoM implants doing high intensity activity were 3 times more likely to undergo revision (HR 3.3 CI 1.1–10.1) compared with low activity recipients.

**Table 4:** Level of physical activity after total hip arthroplasty and risk of revision in patients with small-head metal-on-metal (MoM) and non-MoM hip implants

Factor	UCLA activity score continuous	UCLA activity score in 2 categories		UCLA activity score in 3 categories		
		Not high UCLA 1–7	High UCLA 8–10	Low UCLA 1–4	Moderate UCLA 5–7	High UCLA 8–10
**Non-MoM implant, n = 604**						
Physical activity, mean (SD)						
Not revised	5.6 (1.8)					
Revised	6.5 (1.8)					
Revision, n (%)						
No	–	484 (94)	81 (90)	178 (96)	306 (93)	81 (90)
Yes	–	30 (5.9)	9 (10)	7 (3.8)	23 (7.1)	9 (10)
Median years to end of study/revision						
Not revised	–	14.5	12.7	12.3	16.9	12.7
Revised	–	12.7	10.6	12.7	12.7	10.6
Models of HR (CI)						
Unadjusted	1.3 (1.1–1.6)	Ref.	2.0 (0.9–4.2)	Ref.	1.4 (0.6–3.3)	2.5 (0.9–6.8)
Adjusted**^a^**	1.3 (1.0–1.5)	Ref.	1.8 (0.8–3.8)	Ref.	1.3 (0.6–3.2)	2.2 (0.8–6.0)
**MoM implant, n = 369**						
Physical activity, mean (SD)						
Not revised	6.2 (1.9)					
Revised	6.7 (1.7)					
Revision, n (%)						
No		251 (91)	78 (85)	82 (95)	169 (88)	78 (85)
Yes		26 (9.4)	14 (15)	4 (4.7)	22 (12)	14 (15)
Median years to end of study/revision						
Not revised		16.5	17.5	16.4	16.6	17.5
Revised		10.6	9.1	9.3	11.1	9.1
Models of HR (CI)						
Unadjusted	1.2 (1.0–1.4)	Ref.	1.6 (0.8–3.1)	Ref.	2.6 (0.9–7.4)	3.3 (1.1–10.0)
Adjusted **^a^**	1.2 (1.0–1.4)	Ref.	1.6 (0.8–3.0)	Ref.	2.7 (0.9–7.7)	3.3 (1.1–10.1)

a Adjusted for sex and age at THA.

**Figure 4:**
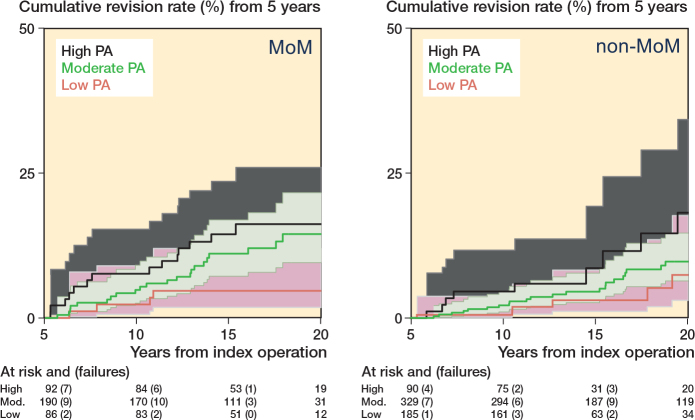
Kaplan–Meier revision events by level of postoperative physical activity in patients with small head metal-on-metal (MoM) and non-MoM implants. 95% CIs of moderate and high level of PA overlaps.

## Discussion

The aim of this study was to explore the risk of revision THA for any cause other than infection associated with moderate- and high-intensity PA over the long-term follow-up. Being physically active postoperatively was associated with an increased risk of revision surgery, especially for those in the most active group (HR 2.7, CI 1.3–5.6). This was an early cohort of patients in whom small-head MoM was relatively common. Therefore, stratified analyses were also conducted by bearing surface, which showed an increased risk of revision for people with MoM implants who reported high levels of PA (HR 3.3, CI 1.1–10.1). These findings must be taken into account alongside recognition of the importance of PA in the prevention of many non-communicable diseases (e.g. cardio-respiratory disease). Active PA likely conveys survival benefits for THA recipients [11]. Importantly also, the youngest and fittest THA recipients are most likely to return to high-intensity PA and more likely to “survive” in a state of good health long enough to be considered for revision surgery.

Current post-THA guidelines formulated by a consensus of surgeons have tended to become less restrictive regarding PA over time, at least in relation to certain activities (e.g., jogging) [5]; however, the size of the long-term association of PA with risk of revision has proved difficult to quantify. Our finding of increased risk of revision in patients doing high levels of PA after THA is consistent with previous studies. A systematic review [12] found evidence of an increased risk of aseptic loosening associated with PA after pooling data from 3 cohort studies. Similarly, other mid- to long-term follow-up studies not only suggested an increased risk of implant failure in patients who were more physically active postoperatively when compared with those less active [13,14], but also that amongst patients who developed femoral osteolysis, the risk of subsequent revision surgery increased with the intensity of PA performed [15].

However, a systematic review exploring the risk of lower limb arthroplasty revision in relation to work and leisure-time physical activities [16] concluded that there was currently only weak evidence for an increased risk of THA revision associated with postoperative PA.

In contrast, other studies have found no relationship between PA and the risk of revision. One case-control study [17], which assessed UCLA activities as a continuous variable, reported no differences in revision rates. Similarly, Ennis et al. found no differences in implant survival between people who engaged in a high or low level of PA at 5 years’ follow-up [18]. Another 2 retrospective studies [2,19] comprising over 2,000 THA recipients reported no greater risk of hip revision amongst active patients compared with those less active.

It is interesting to consider why some studies, but not all, have found an increased risk of revision surgery associated with high levels of PA. First, there are many tools available with which to measure self-reported PA among adults [20] but, despite their ubiquity, a systematic review found that, amongst 85 questionnaires reviewed, the vast majority had at best poor evaluation of their measurement properties including construct validity, content validity, reliability, or responsiveness. Of course, poor measurement of PA could hinder detection of important associations [21]. Amongst the plethora of tools, the UCLA is one that is very commonly used. However, even comparing the studies on this topic using the same tool, different cut-off scores were employed by researchers to categorize UCLA scores. This too would have implications for results. Second, the methods of the studies investigating this research question varied, including, for example, having different durations of postoperative follow-up and different types of statistical analysis, with and without adjustment for different potentially important confounders. Finally, as stated earlier, it is important to bear in mind that this is a nuanced research question: the risk of THA is increased by high levels of leisure-time physical activity (amongst women aged < 45 years and men aged 45–59 years [22], as has been shown for sports participation [23], and it is likely that those who were most active preoperatively will have the highest expectations of participation in exercise/sport postoperatively. Furthermore, fulfilling health-enhancing PA guidelines will convey benefit in terms of reduced morbidity and mortality so that those who undertake higher levels of PA postoperatively are more likely to seek revision arthroplasty if their mobility becomes restricted by pain or dysfunction of their primary THA.

### Limitations

First, comparison of baseline characteristics between THA recipients with and without UCLA scores showed that non-respondents were more likely to be men, slightly younger, smokers, and with higher ASA score at baseline. This suggests some sampling bias with omission of data from a group of people who were less fit and likely less physically active. However, even though this may have resulted in overestimating the levels of PA, this selection bias is unlikely to have affected the demonstrated association between PA and revision. Second, 27% of participants reported different UCLA scores at different time points. For the most part they became less active over time, but a small number reported that they became more active over time. Thus a sensitivity analysis was performed excluding all those who reported changed activity scores. The main findings remained unchanged in this analysis, but the risk estimates obtained had wider confidence intervals. As described earlier, the UCLA activity scale was not available when the registry was first commenced in 1996. For the purposes of this study, an individual was included if there was a UCLA score at least once, but in some cases the first score that was obtained was at the 10-year follow-up. It is plausible therefore that the level of PA reported for these participants would be less than had previously been the case. Importantly, however, these participants have been incorrectly characterized as having done less intense PA, which should have reduced our ability to see a difference in the risk between the less intense and more intense PA groups, biasing our findings. An additional sensitivity analysis was undertaken to explore the effect of 20% of participants having 3 or 4 UCLA scores by including number of scores as a confounder in the model. Once again, similar estimates were obtained (data not shown). Risk of revision is also dependent on surgical factors not accounted for in the analysis, e.g., surgical approach [24]; however, in our sample, neither type of fixation nor size of femoral head affected the outcome (data not shown). Lastly, in a small proportion of revision cases (9–15%) the outcome may not be attributed to PA. Our cumulative risks of revision at 10 and 15 years were relatively low. Revision procedures performed for infection were not included in the outcome as the relation between revisions for infection and postoperative PA is likely confounded by BMI and comorbidities. Patients may decide not to undergo revision surgery, or those at risk of poorer outcomes are not always offered revision surgery.

### Conclusion

We showed that higher levels of PA and moderate levels relative to low levels of PA 10–15 years post THA may increase the risk of revision surgery for any cause.

In perspective, although our findings indicate higher revision risk in patient with high PA, people need to be encouraged to keep active depending on physical capability. It is important to provide high-quality advice preoperatively to younger patients because they tend to be more physically active and have higher expectations in relation to leisure activities or sports that may be associated with an increased revision risk [25].

## Notes

AL, KWB, and EZ designed the study. SD, GN, and EZ analyzed the data, and a first draft of the manuscript was written by EZ. All the authors contributed to the content of the manuscript and approved the final version.

Handling co-editors: Keijo Mäkelä and Robin Christensen

*Acta* thanks Martin Beck and Ola Rolfson for help with peer review of this manuscript.

## References

[1] Pabinger C, Geissler A. Utilization rates of hip arthroplasty in OECD countries. Osteoarthritis Cartilage 2014; 22(6): 734-41. doi: 10.1016/j.joca.2014.04.009. 24780823

[2] Ponzio D Y, Rothermel S D, Chiu Y F, Stavrakis A I, Lyman S, Windsor R E. Does physical activity level influence total hip arthroplasty expectations, satisfaction, and outcomes? J Arthroplasty 2021; 36(8): 2850-7. doi: 10.1016/j.arth.2021.03.052. 33875289

[3] Lübbeke A, Zimmermann-Sloutskis D, Stern R, Roussos C, Bonvin A, Perneger T, et al. Physical activity before and after primary total hip arthroplasty: a registry-based study. Arthritis Care Res (Hoboken) 2014; 66(2): 277-84. doi: 10.1002/acr.22101. 23925916

[4] Hoorntje A, Janssen K Y, Bolder S B T, Koenraadt K L M, Daams J G, Blankevoort L, et al. The effect of total hip arthroplasty on sports and work participation: a systematic review and meta-analysis. Sports Med 2018; 48(7): 1695-726. doi: 10.1007/s40279-018-0924-2. 29691754 PMC5999146

[5] Thaler M, Khosravi I, Putzer D, Siebenrock K A, Zagra L. Return to sports after total hip arthroplasty: a survey among members of the European Hip Society. J Arthroplasty 2021; 36(5): 1645-54. doi: 10.1016/j.arth.2020.11.009. 33277143

[6] Herberts P, Malchau H. Long-term registration has improved the quality of hip replacement: a review of the Swedish THR Register comparing 160,000 cases. Acta Orthop Scand 2000; 71(2): 111-21. doi: 10.1080/000164700317413067. 10852315

[7] Lübbeke A, Baréa C, Miozzari H, Garavaglia G, Gonzalez A, Zingg M, et al. Lessons learnt from 25 years of an institutional hip and knee arthroplasty registry. Rev Med Suisse 2021; 17(763): 2161-5. 34910401

[8] Zahiri CA, Schmalzried T P, Szuszczewicz E S, Amstutz H C. Assessing activity in joint replacement patients. J Arthroplasty 1998; 13(8): 890-5. doi: 10.1016/s0883-5403(98)90195-4. 9880181

[9] Austin P C. Balance diagnostics for comparing the distribution of baseline covariates between treatment groups in propensity-score matched samples. Stat Med 2009; 28(25): 3083-107. doi: 10.1002/sim.3697. 19757444 PMC3472075

[10] Smith A J, Dieppe P, Vernon K, Porter M, Blom A W. Failure rates of stemmed metal-on-metal hip replacements: analysis of data from the National Joint Registry of England and Wales. Lancet 2012; 379(9822): 1199-204. doi: 10.1016/s0140-6736(12)60353-5. 22417410

[11] Warburton D E, Nicol C W, Bredin S S. Health benefits of physical activity: the evidence. CMAJ 2006; 174(6): 801-9. doi: 10.1503/cmaj.051351. 16534088 PMC1402378

[12] Cherian J J, Jauregui J J, Banerjee S, Pierce T, Mont M A. What host factors affect aseptic loosening after THA and TKA? Clin Orthop Relat Res 2015; 473(8): 2700-9. doi: 10.1007/s11999-015-4220-2. 25716213 PMC4488212

[13] Kilgus D J, Dorey F J, Finerman G A, Amstutz H C. Patient activity, sports participation, and impact loading on the durability of cemented total hip replacements. Clin Orthop Relat Res 1991; (269): 25-31. PMID: 1864047 1864047

[14] Ollivier M, Frey S, Parratte S, Flecher X, Argenson J N. Does impact sport activity influence total hip arthroplasty durability? Clin Orthop Relat Res 2012; 470(11): 3060-6. doi: 10.1007/s11999-012-2362-z. 22535588 PMC3462849

[15] Lübbeke A, Garavaglia G, Barea C, Stern R, Peter R, Hoffmeyer P. Influence of patient activity on femoral osteolysis at five and ten years following hybrid total hip replacement. J Bone Joint Surg Br 2011; 93(4): 456-63. doi: 10.1302/0301-620x.93b4.25868. 21464482

[16] Zaballa E, Harris E C, Cooper C, Linaker C H, Walker-Bone K. Risk of revision arthroplasty surgery after exposure to physically demanding occupational or leisure activities: a systematic review. PLoS One 2022; 17(2): e0264487. doi: 10.1371/journal.pone.0264487. 35226696 PMC8884506

[17] Delfin I, Persson G, Ekvall Hansson E. Does physical activity affect risk of revision of total hip arthroplasty? A matched pairs study. Eur J Physiother 2017; 19(3): 124-30. doi: 10.1080/21679169.2017.1296889.

[18] Ennis H E, Lamar K T, Johnson R M, Phillips J L, Jennings J M. Comparison of outcomes in high versus low activity level patients after total joint arthroplasty. J Arthroplasty 2023. doi: 10.1016/j.arth.2023.06.031. 37380140

[19] Crawford D A, Adams J B, Hobbs G R, Morris M J, Berend K R, Lombardi A V Jr. Does activity level after primary total hip arthroplasty affect aseptic survival? Arthroplasty Today 2021; 11: 68-72. doi: 10.1016/j.artd.2021.07.005 34471662 PMC8387823

[20] van Poppel M N, Chinapaw M J, Mokkink L B, van Mechelen W, Terwee C B. Physical activity questionnaires for adults: a systematic review of measurement properties. Sports Med 2010; 40(7): 565-600. doi: 10.2165/11531930-000000000-00000. 20545381

[21] Powell K E, Thompson P D, Caspersen C J, Kendrick J S. Physical activity and the incidence of coronary heart disease. Annu Rev Public Health 1987; 8: 253-87. doi: 10.1146/annurev.pu.08.050187.001345. 3555525

[22] Johnsen M B, Hellevik A I, Baste V, Furnes O, Langhammer A, Flugsrud G, et al. Leisure time physical activity and the risk of hip or knee replacement due to primary osteoarthritis: a population based cohort study (The HUNT Study). BMC Musculoskelet Disord 2016; 17:86. doi: 10.1186/s12891-016-0937-7. 26879518 PMC4754866

[23] Michaëlsson K, Byberg L, Ahlbom A, Melhus H, Farahmand B Y. Risk of severe knee and hip osteoarthritis in relation to level of physical exercise: a prospective cohort study of long-distance skiers in Sweden. PLoS One 2011; 6(3): e18339. doi: 10.1371/journal.pone.0018339. 21479136 PMC3068188

[24] Kuijpers M F L, Hannink G, Vehmeijer S B W, van Steenbergen L N, Schreurs B W. The risk of revision after total hip arthroplasty in young patients depends on surgical approach, femoral head size and bearing type; an analysis of 19,682 operations in the Dutch arthroplasty register. BMC Musculoskelet Disord 2019; 20(1): 385. doi: 10.1186/s12891-019-2765-z. 31438921 PMC6706879

[25] Lützner C, Postler A E, Druschke D, Riedel R, Günther K P, Lange T. Ask patients what they expect! A survey among patients awaiting total hip arthroplasty in Germany. J Arthroplasty 2022; 37(8): 1594-601.e4. doi: 10.1016/j.arth.2022.03.067. 35341925

